# Thromboelastometric Analysis of Anticancer *Cerrena unicolor* Subfractions Reveal Their Potential as Fibrin Glue Drug Carrier Enhancers

**DOI:** 10.3390/biom11091263

**Published:** 2021-08-24

**Authors:** Dawid Stefaniuk, Tomasz Misztal, Mateusz Pięt, Adrian Zając, Magdalena Kopycińska, Anna Matuszewska, Marta Ruminowicz-Stefaniuk, Łukasz Matuszewski, Natalia Marcińczyk, Anna Belcarz, Jerzy Żuchowski, Ilona Skrabalak, Marcin Grąz, Beata Ciołek, Roman Paduch, Magdalena Jaszek

**Affiliations:** 1 Department of Biochemistry and Biotechnology, Institute of Biological Sciences, Maria Curie-Skłodowska University, 20-033 Lublin, Poland; anna.matuszewska@poczta.umcs.lublin.pl (A.M.); ruminowicz@poczta.umcs.lublin.pl (M.R.-S.); ilonaskrabalak123@gmail.com (I.S.); graz@poczta.umcs.lublin.pl (M.G.); beata.rola@poczta.umcs.lublin.pl (B.C.); magdalena.jaszek@poczta.umcs.lublin.pl (M.J.); 2 Department of Physical Chemistry, Faculty of Pharmacy, Medical University of Bialystok, 15-089 Białystok, Poland; 3 Department of Virology and Immunology, Institute of Biological Sciences, Maria Curie-Skłodowska University, 20-033 Lublin, Poland; piet.mateusz@poczta.umcs.lublin.pl (M.P.); makopycinska@gmail.com (M.K.); rpaduch@poczta.umcs.lublin.pl (R.P.); 4 Department of Functional Anatomy and Cytobiology, Institute of Biological Sciences, Maria Curie-Skłodowska University, 20-033 Lublin, Poland; adrian.zajac@poczta.umcs.lublin.pl; 5 Department of Paediatric Orthopaedics and Rehabilitation, Faculty of Medicine, Medical University of Lublin, 20-093 Lublin, Poland; lukasz.matuszewski@umlub.pl; 6 Department of Biopharmacy, Faculty of Pharmacy, Medical University of Bialystok, 15-089 Białystok, Poland; natalia.marcinczyk@umb.edu.pl; 7 Chair and Department of Biochemistry and Biotechnology, Faculty of Pharmacy, Medical University of Lublin, 20-093 Lublin, Poland; annabelcarz@umlub.pl; 8 Department of Biochemistry, Institute of Soil Science and Plant Cultivation—State Research Institute, 24-100 Puławy, Poland; jzuchowski@iung.pulawy.pl

**Keywords:** *Cerrena unicolor*, CCD 841 CoTr, HT-29, fibrin coagulation, fibrin-based sealant, low molecular weight subfractions

## Abstract

In this study, the influence of two subfractions (with previously proven anti-cancer properties) isolated from wood rot fungus *Cerrena unicolor* on the formation of a fibrin clot was investigated in the context of potential use as fibrin glue and sealant enhancers and potential wound healing agents. With the use of ROTEM thromboelastometry, we demonstrated that, in the presence of fibrinogen and thrombin, the S6 fraction accelerated the formation of a fibrin clot, had a positive effect on its elasticity modulus, and enhanced the degree of fibrin cross-linking. The S5 fraction alone showed no influence on the fibrin coagulation process; however, in the presence of fibrin, it exhibited a decrease in anti-proliferative properties against the HT-29 line, while it increased the proliferation of cells in general at a concentration of 100 µg/mL. Both fractions retained their proapoptotic properties to a lesser degree. In combination with the S6 fraction in the ratio of 1:1 and 1:3, the fractions contributed to increased inhibition of the activity of matrix metalloproteinases (MMPs). This may suggest anti-metastatic activity of the combined fractions. In conclusion, the potential of the fractions isolated from the *C. unicolor* secretome to be used as a means of improving the wound healing process was presented. The potential for delivering agents with cytostatic properties introduced far from the site of action or exerting a pro-proliferative effect at the wound site with the aid of a fibrin sealant was demonstrated.

## 1. Introduction

Due to their health-promoting properties, arboreal fungi are an extremely interesting source of bioactive compounds. They exert many types of impact on living organisms, including humans. The compounds have antioxidant [1,2,3,4,5], anti-inflammatory, immunomodulating, antibiotic (antibacterial and antifungal), antiviral, anticancer, antidiabetic [2,6,7], hepatoprotective [8], anti-angiogenic [4,6,9], hypocholesterolomic [10], hemostasis-modulating [11,12,13,14], and wound healing [15] properties. It should be emphasized that many bioactive preparations obtained from fungal biomass or secretome have some or even all these properties.

An appealing species in this context, so far studied mainly as a very efficient source of extracellular laccase, is *Cerrena unicolor* [16,17,18]. This fungus synthesizes low molecular weight secondary metabolites with a broad spectrum of biological activity, confirmed by the results of our recent studies [5,19,20,21]. The results obtained so far have shown antiproliferative [20,22], proapoptotic, and migration-inhibiting [22] properties of low molecular weight subfractions from the *C. unicolor* secretome, especially towards HT-29 colon cancer cells [22]. The need to search for new compounds with effective anticancer properties, high selectivity for cancer cells, and low toxicity towards normal cells is as essential as the search for effective methods of delivering active substances directly to the place of need. This is particularly important for reduction of the risk of complications related to the treatment process, such as the likelihood of metastasis after incomplete resection of organs affected by neoplastic changes.

Fibrin glue (FG) is a commonly used hemostatic agent in treatment of burns, cardiac diseases, and pediatric, liver, orthopedic, vascular [23], and colorectal surgeries [24,25]. It is produced through fibrinogen hydrolysis by the proteolytic enzyme thrombin. As a result of this process, an insoluble fibrin fiber clot is formed, thereby promoting haemostasias, tissue adhesion, and sealing. FG is characterized by good biocompatibility and biodegradability. It can also be a good carrier of antibiotics, genes, growth factors, and cytostatics [26]. The use of a fibrin sealant in surgical procedures, including surgical anastomosis, reduces the costs and treatment time associated with postoperative complications, including reoperation in the case of anastomotic leakage [24]. A combination of FG and oxaliplatin (OXP) enhanced the antitumor performance of OXP in a subcutaneous and abdominal metastasis model of murine colorectal cancer [26]. FG was also shown to reduce adverse effects of intraperitoneally administered 5-FU (5-fluoro uracil) on the healing of anastomoses in rats [27,28].

In the present study, the feasibility of the use of FG supplemented with two low molecular weight antitumor agents of fungal origin as a potential sustainable and safe drug delivery system was investigated for the first time. Two fractions showing the most promising activity inhibiting the proliferation of HT 29 tumor cells [22] were used. One of the fractions also exhibited the ability to promote coagulation in human plasma. The physical properties of the fibrin clot formed were investigated using thromboelastometry and confocal microscopy.

## 2. Materials and Methods

### 2.1. Fungal Culture and Bioactive Fraction Acquisition

The procedure for growing biological material and obtaining bioactive fractions has been described in detail elsewhere [22]. Briefly, the post-culture fluid of *C. unicolor* (*Bull. ex Fr.*) *Murr.* strain FCL 139 (GenBank Accession: DQ056858) idiophasic culture grown on a Lindeberg–Holm medium was ultrafiltered through a 10 kDa cutoff ultrafiltration membrane (EMD Millipore™ Prep/Scale Spiral-Wound Ultrafiltration Modules: TFF-2). Subsequently, the fraction with a molecular weight below 10 kDa was concentrated by reverse osmosis on the TFC-75F membrane (Aquafilter Inc. Hunt Valley, MD, USA) and lyophilized. The preparation was dissolved in water and fractionated by gel permeation chromatography (Sephadex G-15) and six subfractions containing compounds with a molecular weight not exceeding 1.5 kDa were obtained. More detailed information about the biochemical and biological properties of the obtained fractions can be found in the work by Matuszewska et al. [22].

### 2.2. Thromboelastometric Measurements of the Formation Kinetics and Viscoelastic Properties of Fibrin Clots

Thromboelastometric measurements were performed using the ROTEM^®^ Delta rotational thromboelastometric system (Tem International GmbH, Manheim, Germany) [29]. Samples of human fibrinogen (25 mg/mL) were incubated at room temperature with saline (control) or with fraction S6 (30–1000 μg/mL), S5 (100 μg/mL), or a combination of both fractions (S6 in concentration of 30, 100, 300, 600, 1000 μg/mL and constant S5 concentration of 100 μg/mL). After 10 min of incubation, the samples were analyzed to evaluate the kinetics of formation and the viscoelastic properties of fibrin clots. All ROTEM^®^ coagulation measurements were performed by the same experienced operator as follows: a 320 µL sample was transferred into a preheated (37 °C) cup containing 20 μL of activating mix (1.7 U/mL of bovine thrombin in 0.17 M calcium chloride) and repeatedly pipetted gently to mix the components (final concentrations of the activators in the samples: 0.1 U/mL of thrombin and 10 mM calcium chloride). The parameters characterizing clotting initiation (CT, clotting time; CFT, clot formation time), propagation (alpha angle; MaxV, maximal velocity of clot formation; MaxV-t, time to reach maximal velocity of clotting), and stabilization (MCF, maximum clot firmness; G, shear elastic modulus strength) were measured.

### 2.3. Confocal Microscopy of Fibrin Clots

Samples (100 µL) of human fibrinogen (2.5 mg/mL) were pre-incubated for 10 min at room temperature with saline (control) or with fractions S6 (30–300 µg/mL), S5 (100 µg/mL), or a combination of both fractions. After that, the samples were supplemented with Alexa Fluor 488-labelled human fibrinogen (0.15 µM final conc., approximately 15 dye molecules for each fibrinogen molecule) and preincubated for 2 min at 37 °C. Next, CaCl_2_ + bovine thrombin (10 mM and 0.1 U/mL final conc., respectively) was added, the mixture was mixed vigorously, and 25 µL aliquots were transferred to microchamber slides (Ibidi µ-slide VI; Animalab). The samples were protected from light and incubated for 2 h at 37 °C in humid atmosphere. After the coagulation time, the architecture of the clots was imaged using a fluorescence microscope with the confocal imaging system Zeiss Axio Examiner Z.1 (Carl Zeiss Microscopy GmbH, Jena, Germany) with a confocal scanning unit Yokogawa CSU-X1 (Yokogawa Electric Corporation, Japan) equipped with a high speed digital camera CCD C9300-221 (Hamamatsu Photonics K.K., Japan) and a W Plan-Apochromat 20×/1.0 water immersion objective (Carl Zeiss Microscopy GmbH, Jena, Germany). At least 10 pictures of different areas of each clot were taken. One representative image is presented. The relative fluorescence intensity (associated with fiber density) was quantified using SlideBook 6 software.

### 2.4. Thrombin Activity Assay

To measure the effect of the S6 fraction on thrombin activity, 40 µL aliquots of samples (composed of an appropriate, 30–1000 µg/mL, concentration of the S6 fraction (or saline in the control) and bovine thrombin in 50 mM Tris-HCl, 0.1 M NaCl, 2 mM CaCl_2_, 0.5% BSA, pH 7.5 buffer) incubated for 10 min at room temperature were transferred into microplate wells containing 197.5 µL of the buffer. After mixing and incubating at 37 °C for 3 min, the samples were supplemented with 12.5 µL of 4 mM S-2238 (chromogenic substrate for thrombin). The absorbance of the released color product was recorded for 10 min with stirring at a wavelength of 405 nm using a Sunrise plate reader (Tecan, Männedorf, Switzerland).

### 2.5. Cell Cultures

All cell assays—i.e., MTT, NR, and Hoechst 33342/PI staining, zymography, and immunoblotting—were performed on cells, medium supernatants, or cell lysates of two lines: human colon cancer cells HT-29 (ATCC HTB-38) cultured in RPMI 1640 medium and human normal colon epithelial cells CCD 841 CoTr (ATCC CRL-1807) grown on a mixture of DMEM and RPMI 1640 media (1:1) supplemented with 10% (*v*/*v*) FBS, 100 U/mL penicillin, and 100 μg/mL streptomycin. The cells were kept at 37 °C (HT-29) or 34 °C (CCD 841 CoTr) in a humidified atmosphere with 5% CO_2_.

### 2.6. Fibrinogen and Fraction Preparation for In Vitro Cell Analyses

For the in vitro cell analyses, fibrinogen was dissolved in appropriate culture medium (RPMI or DMEM:RPMI) to the concentration of 50 mg/mL. For the experiments, the solution was diluted to appropriate concentrations. Based on screening results (Figure S2), the concentration of 2.5 mg/mL of fibrinogen was selected for the further analyses on the cell cultures. Freeze-dried fractions S5 and S6 were dissolved in appropriate culture medium to obtain a stock solution of 4 mg/mL and a final concentration (30, 100, and 300 µg/mL) used in the assay; additionally, to test the potential interaction effect, fractions S5 and S6 were mixed at 3:1, 1:1, and 1:3 ratios.

### 2.7. MTT Assay

The MTT (methylthiazolyldiphenyl-tetrazolium bromide) assay is a colorimetric test to assess the metabolic activity of cells. The activity of NAD (P)-dependent oxidoreductases reflects the number of viable cells. These enzymes are capable of reducing tetrazolium dye MTT to its purple insoluble formazan derivative. The aim of the analysis is to present the level of proliferation after treatment with antiproliferative substances tested [30,31]. In brief, 1 mL of cell suspensions were seeded on 24-well plates (1 × 10^5^ cells/mL) and incubated for 24 h to adhere. Afterwards, the medium was discarded, and the studied solutions were added (30, 100, and 300 µg/mL of final concentration). In variants with fibrinogen, 1 U/mL of bovine thrombin was added to allow clotting. Concentrations of fibrinogen ranging from 0 to 25 mg/mL were used to determine the level of inhibition of cytotoxicity and proliferation by fibrinogen. The concentration of 2.5 mg/mL was used for further analyses of the influence of the fractions due to its low negative effect on cell proliferation in vitro and value close to the physiological one. After 24 or 96 h, the clots were removed, and the procedure of the MTT assay was performed as described in [22,32].

### 2.8. NR Assay

The NR assay is based on changes in cell membrane integrity. Neutral red is absorbed by living cells and accumulated in “acidic” organelles, such as lysosomes. After rinsing, the dye is removed from dead cells. In an acidified environment, this dye is released from living cells and the amount of live cells is proportional to the amount of dye released [33,34]. The procedure was carried out similarly as described [22,32]. Cell lines and samples were prepared as in the MTT method described above. The incubation time of the cells with the fractions and the fibrin clot was 24 h.

### 2.9. Morphological Identification of Apoptotic and Necrotic Cells

Assessment of apoptosis and necrosis in cultures was performed using DNA binding dyes—Hoechst 33342 and propidium iodide (PI). The analyses were performed under an Axiovert 200M confocal microscope with an LSM 5 PASCAL scanning head (Zeiss, Jena, Germany). Cell cultures for the analysis were carried out in a 35 × 10 mm tissue culture dish (SPL Life Sciences, Pocheon, South Korea) and inoculated with 2 mL of a cell suspension at a concentration of 1 × 10^5^ cells/mL. After 24 h incubation with the test fractions at a final concentration of 300 μg/mL and 2.5 mg/mL fibrinogen, 5 μL of Hoechst 33342 (0.4 mg/mL) and PI (0.5 mg/mL) mixed in a 2:3 ratio were added where applicable. The cultures were incubated for 5 min at 37 °C in the dark. After this time, the medium with the staining solution was removed and replaced with PBS buffer with Ca^2+^ and Mg^2+^ ions. The buffer exchange was followed by microscopic observations at an excitation wavelength of λ = 420 nm. The number of apoptotic and necrotic cells was calculated based on the observation of at least 1000 cells in randomly selected fields of view. Cells exhibiting blue fluorescence of fragmented nuclei were interpreted as apoptotic, whereas cells with pink fluorescence of whole nuclei were classified as necrotic. Each analysis was repeated three times [22,35].

### 2.10. Evaluation of the MMP Activity—Gelatin Zymography

The cells were cultured on 24-well plates and incubated for 24 h with the studied fractions (at 300 µg/mL), with or without addition of 2.5 mg/mL fibrinogen. Afterwards, media from each variant were collected and stored at −80 °C. Zymography was performed as published previously by Matuszewska et al. 2019 [22]. The obtained bands were measured densitometrically using ImageJ 1.51 software (National Institute of Mental Health, Bethesda, MD, USA).

### 2.11. Evaluation of the MMP Activity—Immunoblotting Assay

The control and tested cells (incubated with subfractions at 300 µg/mL) grown in 6-well cell culture plates (Corning, New York, NY, USA) were mechanically harvested on ice with a laboratory scraper and transferred to fresh chilled laboratory tubes, which were then centrifuged at 12,000× *g* and 4 °C for 10 min. After the centrifugation step, the supernatant was discarded, and the cell pellet was resuspended in cold RIPA buffer, boiled in a water bath for 10 min, and centrifuged (12,000× *g*, 20 min). The obtained supernatants were used for further analyses. The protein concentration in the extracts of the control and test cells was determined according to the Bradford method [36]. Unidirectional protein electrophoresis on SDS-PAGE polyacrylamide gel (12%) was performed according to the Laemmli method [37]. Each sample contained 80 µg/mL of proteins. After electrophoresis, separated proteins were transferred onto PVDF Immobilon P membranes with a 0.45 μm pore size. The PVDF membranes with proteins were incubated for one hour in a 5% milk solution to block incorporation of non-specific antibodies and then incubated overnight with a solution of anti-MMP-2 (1:500; Santa Cruz Biotechnology Inc., Dallas, TX, USA) and anti-MMP-9 (1:500; Invitrogen, Thermo Fisher Scientific, Waltham, MA, USA) primary antibodies at 4 °C. The next day, primary antibodies were discarded, and the membranes were incubated for 2 h at room temperature with alkaline phosphatase-conjugated secondary antibodies (1:10000). To visualize proteins, solutions of alkaline phosphatase substrates were used: NBT (nitrotetrazolium blue), 9 mg in 300 μL H_2_O and 700 μL DMF (N,N-dimethylformamide, Sigma) and BCIP (5-Bromo-4-chloro-3-indolyl phosphate, Sigma), 4.5 mg in 1 mL DMF. The membranes were incubated in the substrate mixture for approximately 10 min, washed with distilled water, and dried at room temperature. The obtained fringes were measured densitometrically using ImageJ 1.51 software (National Institute of Mental Health, Bethesda, MD, USA) [38,39].

### 2.12. Statistical Analysis

All results obtained are the mean ± SD from a minimum of three experiments performed in triplicate. Statistically significant differences were calculated using one-way ANOVA and post hoc Dunnett’s test.

## 3. Results

### 3.1. Enhancement of Formation Dynamics and Viscoelastic Properties of the Fibrin Clot

**Figure 1:**
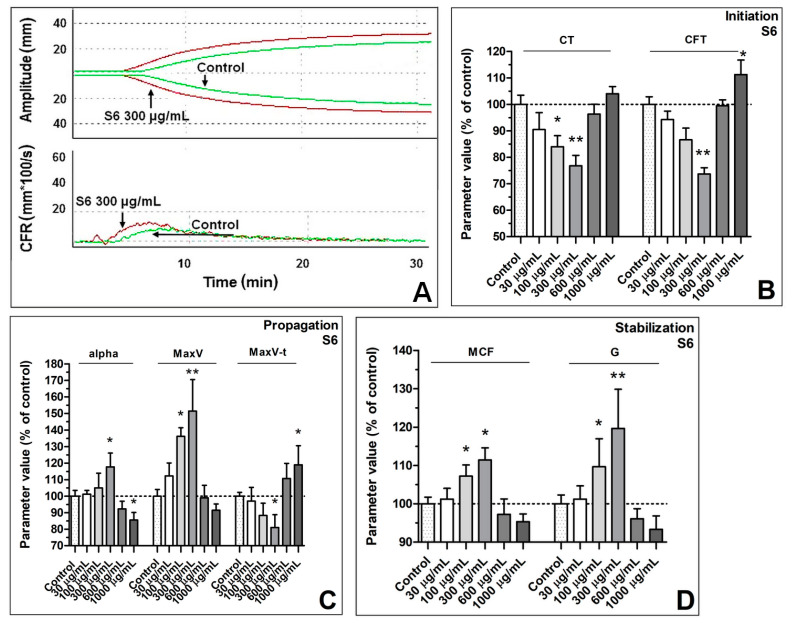
Effect of fraction S6 on the formation kinetics and viscoelastic properties of fibrin clot. Samples of human fibrinogen were incubated without (control) or with the indicated concentrations of S6 for 10 min at room temperature. Next, clotting was initiated by mixing the samples with the activating mix containing bovine thrombin and CaCl_2_ (final concentrations of activators: 0.1 U/mL and 10 mM, respectively), and the formation kinetics and viscoelastic properties of the clot were determined using rotational thromboelastometry. Representative coagulation profiles (showing increase of clot firmness over time, the upper part) and record of the clot formation rate (CFR, the distribution of the velocity of clot formation in time, lower part) from one experiment (out of 7) are presented for control sample and selected S6 concentration in panel **A**. (**B**–**D**) Parameters associated with clotting initiation (CT, clotting time; CFT, clot formation time), propagation (alpha angle; MaxV, maximal velocity of clot formation; MaxV-t, time to reach maximal velocity of clotting), and stabilization (MCF, maximum clot firmness; G, shear elastic modulus strength) were measured. Data are means ± SD from *n* = 7 experiments The parameter value range (and mean value ± SD) in the control was CT: 254–363 s (309 ± 52 s); CFT: 210–276 s (243 ± 33 s); Alpha: 34–40 degrees (37 ± 3 degrees); MaxV: 3–5 mm*100/s (4 ± 1 mm*100/s); MaxV-t: 392–468 s (430 ± 38 s); MCF: 22–26 mm (24 ± 2 mm); G: 1410–1756 (1583 ± 172); * *p* < 0.05; ** *p* < 0.01.

At clotting initiation (Figure 1B), fraction S6 shortened the CT (clotting time) by 17 ± 5% at the concentration of 100 µg/mL and by 24 ± 5% at the concentration of 300 µg/mL; the higher concentrations of S6 did not reduce the CT significantly. The CFT (clot formation time) parameter at the concentration of 300 µg/mL was reduced by 26 ± 5%, 600 µg/mL did not change the parameter significantly, and the concentration of 1000 µg/mL extended CFT by 12% ± 6 compared with the control.

**Figure 2:**
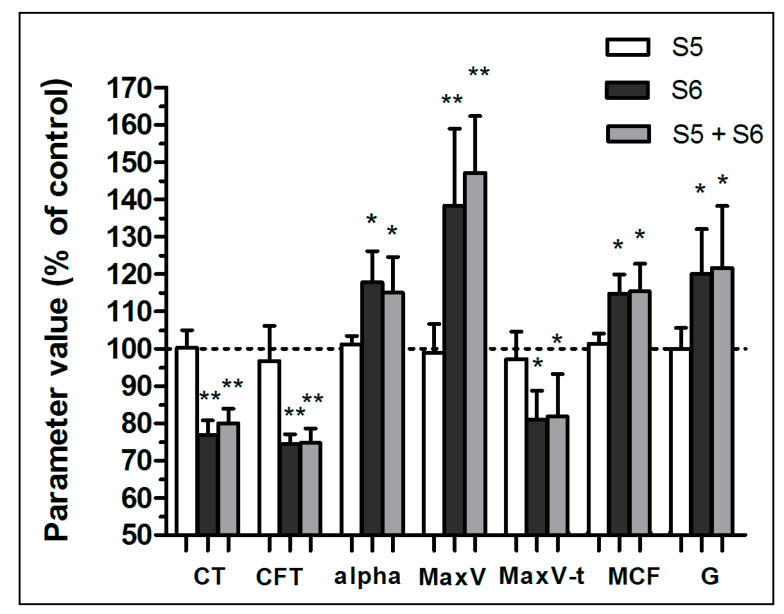
Verification of the interaction effect of fractions S5 (100 µg/mL) and S6 (300 µg/mL) on procoagulant activity. Parameters associated with clotting initiation (CT, clotting time; CFT, clot formation time), propagation (alpha angle; MaxV, maximal velocity of clot formation; MaxV-t, time to reach maximal velocity of clotting), and stabilization (MCF, maximum clot firmness; G, shear elastic modulus strength) were measured. Data are means ± SD from *n* = 7 experiments, * *p* < 0.05; ** *p* < 0.01.

Summarizing, fraction S6 in a concentration range of 30–300 µg/mL produced dose-dependent augmentation of the fibrin clot formation dynamics (Figure 1A). This was manifested in a decrease in the CT and CFT values (indicating faster initiation of fibrin formation, Figure 1B), elevation of alpha and MaxV, and shortening the time required to obtain MaxV (enhanced clotting propagation, Figure 1C). The parameters of the mechanical stability (MCF) and viscoelasticity (G) of fibrin clots were elevated (Figure 1D). Conversely, the presence of the higher concentrations of S6 (600–1000 µg/mL) in the samples was associated with reduced initiation and propagation of fibrin clot formation and with diminished stability and viscoelasticity of such clots. Fraction S5 per se neither influenced fibrin formation nor modulated S6-evoked augmentation of fibrin formation and viscoelasticity (Figure 2). Fraction S6 also exhibited the ability to promote coagulation in human plasma during screening trials (Supplementary Figure S1).

During the propagation phase (Figure 1C), the S6 fraction at the concentration of 300 µg/mL increased the alpha angle by 17 ± 8%. The concentration of 1000 µg/mL decreased the value of the parameter by 16 ± 4%, whereas the intermediate concentration (600 µg/mL) did not significantly change the alpha parameter.

The maximum velocity (MaxV, expressed as a rate of clot amplitude increase in time), describing the maximum of the first derivative of the clotting curve, was increased by 33 ± 5% at the S6 concentration of 100 µg/mL and by 49 ± 19% at the concentration of 300 µg/mL. The higher S6 concentration did not increase MaxV significantly. The MaxV-t (the time to maximum velocity in seconds counted from test start until the maximum of the first derivative of the curve is reached) was shortened by 22 ± 7% at a concentration of 300 µg/mL. The concentration of 1000 µg/mL extended MaxV-t by 17 ± 10%, while the concentration of 600 µg/mL did not change MaxV-t in significant way.

During the stabilization phase (Figure 1D), 100 µg/mL and 300 µg/mL of S6 increased MCF (maximum clot firmness) by 7 ± 2% and 11 ± 2%, respectively; the higher concentrations of S6 did not change MCF significantly. The shear elastic modulus strength (G parameter) increased by 9 ± 7% and 18 ± 10% in the presence of the 100 µg/mL and 300 µg/mL concentrations, respectively, whereas the higher concentrations of S6 did not change G significantly.

### 3.2. Effect of Fractions on Thrombin Activity

**Figure 3:**
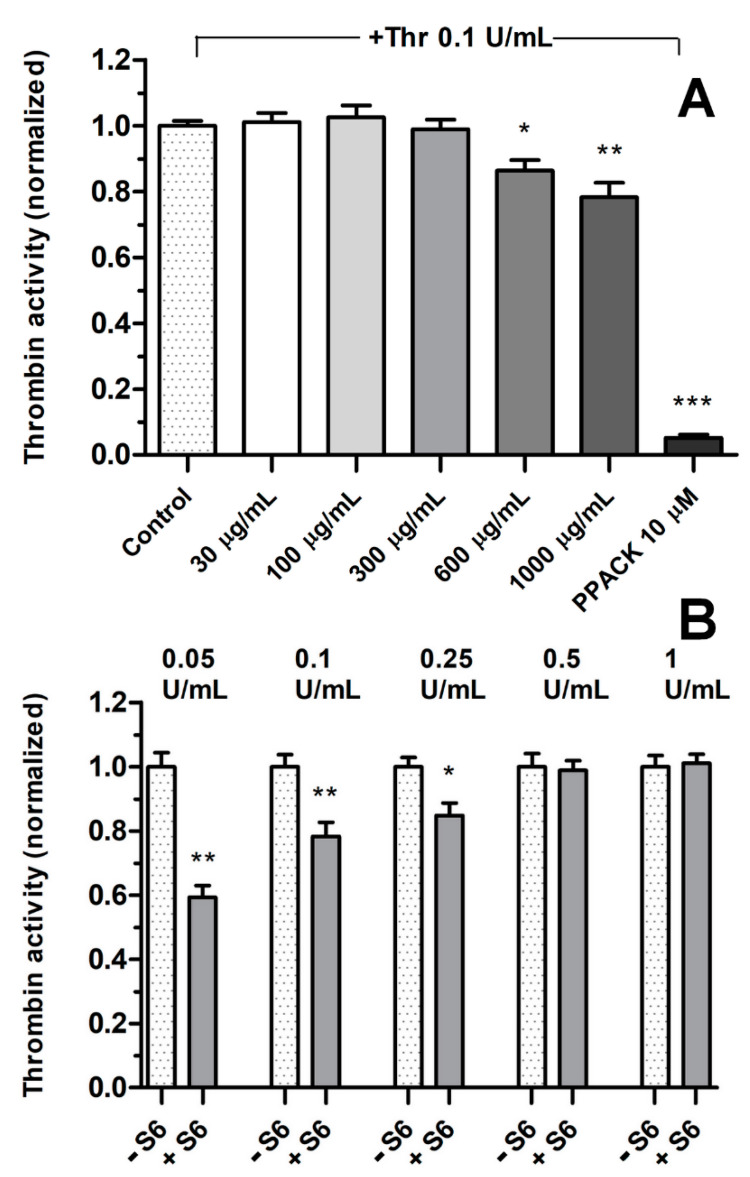
Thrombin activity in the presence of increasing S6 concentrations. Bovine thrombin (0.1 U/mL) was incubated with the indicated increasing concentrations of S6 for 10 min at room temp. This was followed by 2 min incubation at 37 °C in the microplate reader. The reaction was initiated by the addition of chromogenic substrate S-2238 (4 mM) and an increase in absorbance (using 405 nm wavelength) associated with the appearance of the reaction product was measured for 10 min with stirring. The results were normalized to control samples containing vehiculum (saline) instead of S6. The effect of the increasing S6 concentrations on thrombin activity was compared with PPACK (direct thrombin inhibitor) as a positive control (**A**). Samples containing indicated thrombin activities were incubated with saline (control sample, −S6) or with S6 (+S6) at concentration 1 mg/mL (**B**). The results are means ± SD from 5 independent experiments, each in triplicate. * *p* < 0.05; ** *p* < 0.01; *** *p* < 0.001 vs. control.

To investigate whether the augmentation of fibrin formation by the S6 fraction may result from enhancement of thrombin (Thr) activity, we measured its activity in the presence of different S6 concentrations. As shown in Figure 3, concentrations of S6 that were found to stimulate coagulation in the pure fibrinogen + Thr system (30–300 µg) did not affect the activity of Thr at the concentration used in thromboelastometric measurements of the fibrin formation rate (i.e., 0.1 U/mL). Instead, concentrations of S6 above the stimulating range (>300 µg/mL) inhibited Thr activity in a dose-dependent manner. S6 inhibited thrombin activity by 18 ± 3% at the concentration of 600 µg/mL and by 23 ± 4% at the concentration of 1000 µg/mL (Figure 3A). This effect declined along the increasing concentration of Thr, suggesting the presence of a non-competitive inhibitor. The S6 fraction at the concentration of 1000 µg/mL decreased the activity of 0.05 U/mL Thr by 41 ± 3%, 0.1 U/mL by 23 ± 4%, and 0.25 U/mL by 18 ± 3%; at the higher concentrations of Thr, fraction S6 did not show any inhibitory effect (Figure 3B). Fraction S5 (up to 100 µg/mL) did not modulate Thr activity (result not shown).

### 3.3. Confocal Microscopy of the Density of Fibrin Clots

**Figure 4:**
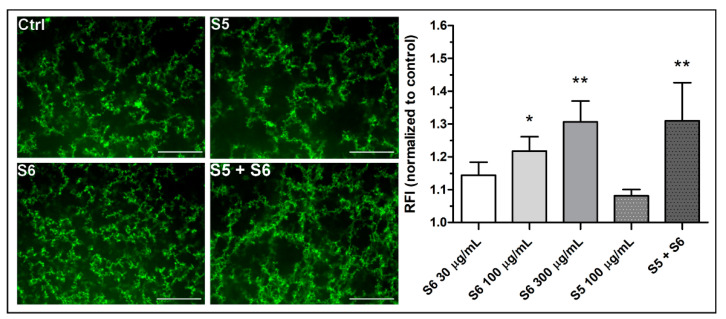
Confocal microscopy and analysis of the relative fibrin clot density. Samples of pure human fibrinogen supplemented with AF488-labelled human fibrinogen (to visualize fibrin) were incubated for 10 min with fractions S6 (30–300 µg/mL) or S5 (100 µg/mL) or with their combinations (300 µg/mL + 100 µg/mL of S6 and S5, respectively). Control samples were supplemented with saline. Clotting was triggered by bovine thrombin. After the gelation point (2h), the clots were analyzed using confocal microscope. Representative fibrin strands from 5 independent experiments are presented. Relative fluorescence intensity (RFI, fluorescence of fibrin in the studied sample in relation to fluorescence of fibrin in the control) was used as a measure of the content of fibrinogen molecules in the analyzed clots, i.e., the extent of polymerization. * *p* < 0.05; ** *p* < 0.01, *n* = 5. The scale bar represents 30 µm.

As indicated by confocal microscopy (Figure 4), the presence of the S6 fraction in samples composed of pure fibrinogen and thrombin was associated with dose-dependent enhancement in the density of fibrin. Fraction S6 increased the density of fibrin fibers by 21 ± 5% at the concentration of 100 µg/mL and by 30% ± 7 at the concentration of 300 µg/mL. The combination of S6 (300 µg/mL) and S5 (100 µg/mL) increased the density by 30 ± 12%. Fraction S5 per se did not produce significant changes in fibrin properties, while the S6-evoked fibrin densification was maintained in the concomitant presence of S5. There was no additive or synergistic effect between S6 300 µg/mL and S5 + S6 groups.

### 3.4. Effect of Fibrinogen on the Cell Viability

The polymerized fibrin alone formed from fibrinogen in concentrations above the physiological value exhibited antiproliferative properties (Figure S2). Such an effect may be an implication of increased osmotic pressure. On the other hand, concentrations lower than 0.5–1 µg/mL were not sufficient for the clot formation. Therefore, the concentration of 2.5 µg/mL was selected for the further in vitro analyses. Furthermore, thrombin alone at 1 U/mL did not affect cell viability at all.

### 3.5. Effect of the S5 and S6 Fractions in Combination with Fibrin on Cell Viability and Proliferation

**Figure 5:**
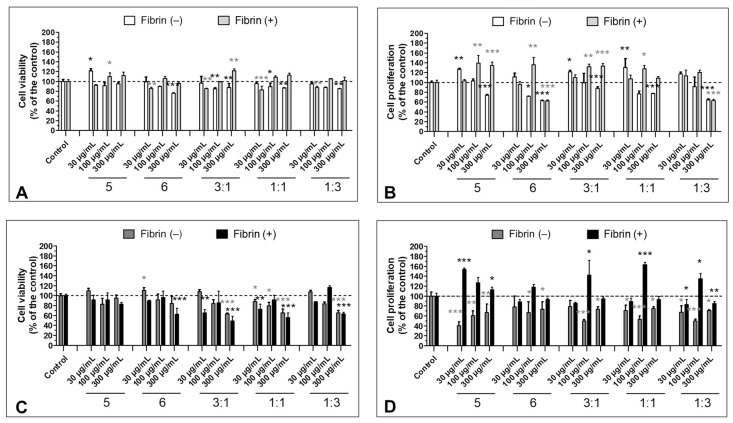
Effect of S5 and S6 on the viability and proliferation of CCD 841 CoTr and HT-29 cells (MTT method). The viability of the CCD 841 CoTr (**A**) and HT-29 (**C**) cells was slightly decreased by the S5 and S6 fractions; moreover, a stronger effect was exerted by the fractions administered in the combinations. The effect was independent of the presence of the fibrin clot. In turn, the proliferation of the normal (**B**) and cancer cells (**D**) was affected by the presence of the clots to a greater extent. Fibrin exerted a protective effect against S5 and S6 fractions, especially those administered at 100 µg/mL. However, the lower (30 µg/mL) and higher (300 µg/mL) concentrations had a strong proliferation-inhibiting effect towards cancer cells and proliferation-inducing effect on normal cells. * *p* < 0.05; ** *p* < 0.01; *** *p* < 0.005; one-way ANOVA, *n* = 3, post hoc: Dunnett’s test.

In the case of HT-29, fractions S5 and S6 administered in 3:1, 1:1, and 1:3 ratios exerted a statistically significant cytotoxic effect, irrespective of the presence of fibrin clots (Figure 5C). On the other hand, longer incubation (96 h) revealed a distinct effect of the polymerized fibrin on the cells. When combined with S5 and S6, fibrin protected cancer cells from the antiproliferative effect of the fractions. However, combined S5 and S6 (3:1, 1:1, 1:3) at concentrations of 30 and 300 µg/mL reduced this effect (decreasing cell proliferation to 85.9 ± 1.8, 83.1 ± 10.2, and 88.8 ± 6.9% of the control by 3:1, 1:1, and 1:3 at 30 µg/mL, respectively, and to 94.5 ± 3.1, 93.2 ± 5.9, and 85.1 ± 4.4% of the control by 3:1, 1:1, and 1:3 at 300 µg/mL, respectively). An enhanced effect was noted at 100 µg/mL (up to 142.4 ± 29.5, 163.0 ± 4.9, and 134.9 ± 10.9% of the control by 3:1, 1:1, and 1:3 ratios, respectively) (Figure 5D). Fibrin administered with S5 and S6 fractions increased proliferation of the CCD 841 CoTr cell as well, with the highest effect of observed at 100 µg/mL (the effect of all fractions was higher than 120% of the control). In contrast with the HT-29 cells, proliferation of the normal cells was induced by the fractions at 30 and 300 µg/mL (excluding S6 at 30 µg/mL and S6 and 1:3 at 300 µg/mL) (Figure 5B). Fractions administered without addition of fibrinogen exerted effects similar to the ones presented earlier by Matuszewska et al. [22]—a mild inhibition or significant increase in proliferation of normal cells and significant inhibition of the proliferation of the cancer cells.

### 3.6. Pro-Apoptotic Activity of S5 and S6 Fungal Fractions

**Figure 6:**
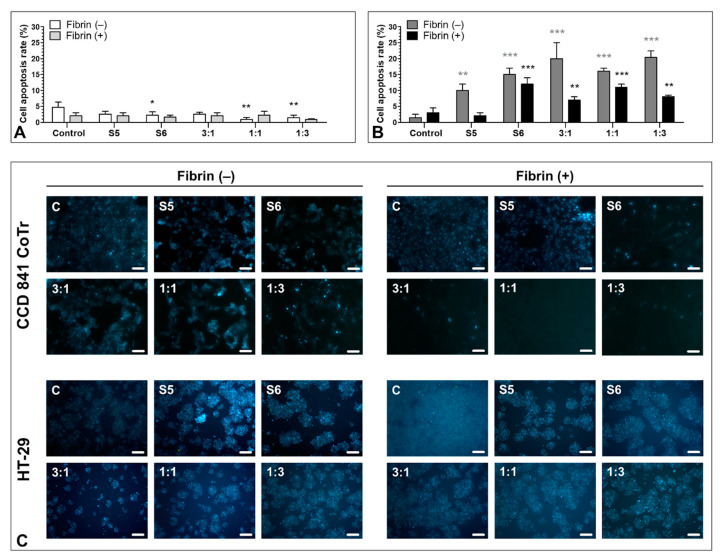
Pro-apoptotic effect of the fractions. Effect of apoptosis induction in the CCD 841 CoTr (**A**) and HT-29 cells (**B**) evaluated with Hoechst 33342/PI staining. (**C**) shows a representative microscopic image of cells influenced by the tested fractions. The studied fractions, administered at 300 µg/mL, exerted a proapoptotic effect, which was stronger in the case of the fraction administered in the 3:1, 1:1, and 1:3 ratios. Fibrinogen exerted an antiapoptotic and protective effect on the HT-29 cells. However, the number of apoptotic cells was still higher than in the control (excluding the S5 fraction). * *p* < 0.05; ** *p* < 0.01; *** *p* < 0.005; one-way ANOVA, *n* = 3, post hoc: Dunnett’s test. The scale bars represent 200 µm.

The evaluation of morphological changes in the normal and colorectal cancer cells (Figure 6) revealed desired pro-apoptotic potential of the studied fractions. No significant increase in apoptosis or necrosis induction was observed in the (CCD 841 CoTr) cells. The apoptotic effect did not exceed 5% in cell cultures without fibrin. Addition of fibrin to the cell cultures caused a slight decrease in the already low apoptosis level.

Analysis of the HT-29 cells with no fibrin matrix revealed that both S5 and S6 fractions exerted a pro-apoptotic effect (an increase in the apoptotic rate from 1.5 ± 1.1% in the control to 10.0 ± 2% and 15.0 ± 2.0% by S5 and S6, respectively). The application of combined fractions was even more efficient in apoptosis induction in the cancer cells (increasing the apoptotic cell rate up to 20.0 ± 5.0, 16.0 ± 1.0, and 20.3 ± 2.1% by 3:1, 1:3, and 1:1, respectively). Fibrin seemed to limit the pro-apoptotic effect of all variants, compared with the control, indicating possible protective activity (a decrease in the apoptotic rate to 2.0 ± 1.0, 12.0 ± 2.0, 7.0 ± 1.0, 11.0 ± 1.0, and 8.0 ± 0.5% after administration of S5, S6, and the combination in 3:1, 1:1, and 1:3 ratios, respectively). However, the apoptotic ratio was still higher than in the control (3.0 ± 1.5%) (Figure 6B). The presence of the fraction also negatively influenced the cell density. This effect was mitigated in the presence of fibrin.

No necrotic effect exceeding 1% was observed in either the control or the test cells of each cell line with or without the fibrin matrix.

### 3.7. Effect of the S5 and S6 Fractions with Fibrinogen on MMP-2 and MMP-9

**Figure 7:**
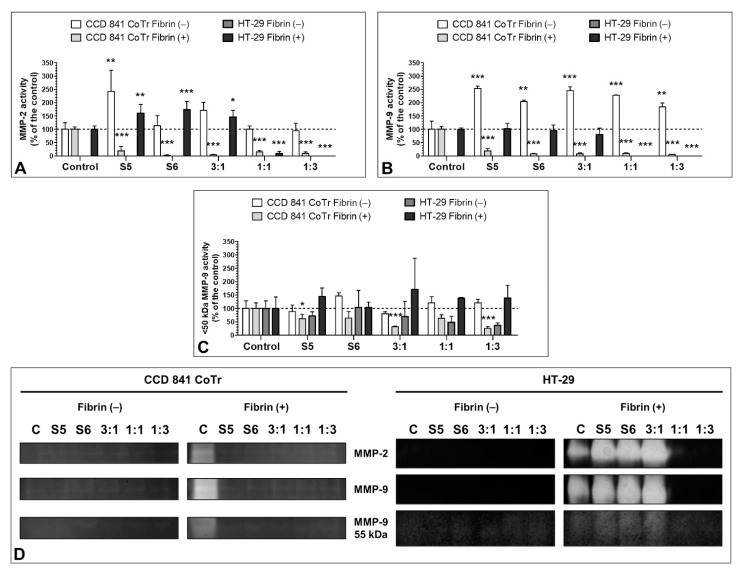
Gelatin zymography of CCD 841 CoTr and HT-29 MMPs in the presence of the S5 and S6 fractions and fibrin matrix. Effect of the S5 and S6 fractions at 300 µg/mL administered individually and in combinations (3:1, 1:1, 1:3) on the activity of MMP-2 (**A**) and MMP-9 (**B**) secreted by the CCD 841 CoTr and HT-29 cells induced with 2.5 mg/mL fibrinogen measured with gelatin zymography method. The results of the activity of MMPs secreted by cells with no fibrin matrix were below the limit of detection (as seen in zymograms on panel (**D**)). Effect of S5 and S6 on the <50 kDa isoform of MMP-9 secreted by the CCD 841 CoTr and HT-29 cells (**C**). The presence of fibrinogen clots induced the activity of MMPs. Furthermore, the activity of MMP-2 was increased by S5, S6, and 3:1 variants, with no effect on the activity of MMP-9. The activity of the <50 kDa isoform of MMP-9 was decreased by all variants, with the strongest effect exerted by the 1:1 and 1:3 fraction ratios. * *p* < 0.05; ** *p* < 0.01; *** *p* < 0.005; one-way ANOVA, *n* = 4, post hoc: Dunnett’s test.

MMP-2 and MMP-9 secreted by HT-29 without fibrin clots exhibited very low activity (the results are not presented, as the bands were too weak to perform densitometric analysis). The enzyme activity was greatly increased in the presence of fibrin clots. Furthermore, S5 and S6 administered individually and in the 3:1 combination induced a significant increase in the activity of MMP-2 (up to 160.0 ± 33.7, 174.1 ±30.7, and 146.6 ± 23.8% of the control, respectively) with no simultaneous effect on MMP-9 activity. However, combinations 1:1 and 1:3 decreased MMP-2 and MMP-9 activity almost completely (Figure 7A,B). Additionally, the activity of the <50 kDa isoform of MMP-9 was demonstrated. The HT-29 cells in the presence and absence of fibrinogen secreted this enzyme form. Its activity was increased in the presence of fibrinogen clots with the strongest effect of the 3:1 ratio (activity increased to 170.9 ± 128.2% of the control) and decreased by S6 and the 1:1 and 1:3 combinations without fibrinogen clots (to 71.6 ± 16.1, 48.5 ± 22.5, and 36.6 ± 10.1% of the control, respectively). However, the values were not statistically significant (Figure 7C,D). Noteworthy, fibrin strongly induced the activity of MMP-2 and MMP-9.

Fractions administered without fibrin significantly increased activity of MMP-2 and MMP-9 secreted by CCD 841 CoTr, with the strongest effect of S5 and 3:1 ratio towards MMP-2 (the activity increased to 243.0 ± 78.8 and 170.5 ± 30.8% of the control, respectively) and all fractions towards MMP-9 (the activity increased about 2-fold), with the strongest influence exhibited by S5 and 3:1 (up to 254.0 ± 8.0 and 244.9 ± 14.3% of the control, respectively). In the control, fibrin significantly increased activity of MMPs, which was mitigated by the fractions (Figure 7A,B).

**Figure 8:**
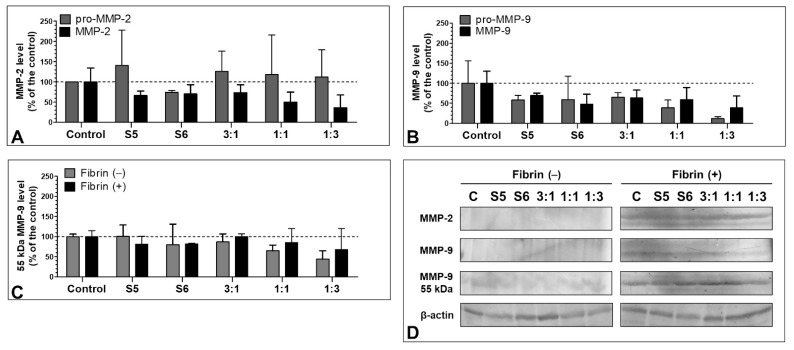
Immunoblotting of MMP-2 and MMP-9 in HT-29 cells. The levels of MMP-2 (**A**) and MMP-9 (**B**) in HT-29 were evaluated with the immunoblotting assay. As in the case of zymography, the level of the enzymes in the fibrin uninduced cells was under the detection threshold. The variants decreased the levels of MMP-2 and MMP-9 active forms and MMP-9 zymogen and caused an increase in the pro-MMP-2 level. Additionally, an isoform of MMP-9 with <50 kDa mass was detected; its level evaluated with immunoblotting (**C**,**D**) was decreased by all studied fractions.

The immunoblotting assay revealed that the presence of fibrin clots in the cell cultures increased the MMP-2 and MMP-9 levels in the HT-29 cells. The level of MMPs was extremely low in all variants cultured without fibrinogen, including the control. In the presence of fibrin, both S5 and S6 fractions administered individually and in combinations decreased the MMP levels, with the highest inhibiting activity of the 1:1 and 1:3 ratios towards MMP-2 (up to 49.6 ± 25.4 and 36.4 ± 31.6% of the control, respectively) as well as S6 and 1:1 towards MMP-9 (47.7 ± 24.7 and 38.8 ± 29.5% compared with the control, respectively) (Figure 8A,B). These results were, however, not statistically significant. Distinct results were obtained in the case of the levels of pro-enzymes. The pro-MMP-2 level was increased by all variants (with the strongest effect of the S5 fraction (up to 141.1 ± 86.9%)) (Figure 8A), while the pro-MMP-9 level was decreased (with the strongest effect exhibited by the 1:1 and 1:3 variants (to 39.4 ± 19.2 and 11.8 ± 4.6% of the control, respectively)) (Figure 8B).

Similar to the zymography, both in the presence and absence of fibrin, an MMP-9 <50 kDa isoform was detected. The strongest inhibiting effect against the enzyme was exhibited by the 1:1 and 1:3 variants in both conditions (to 64.9 ± 14.2 and 44.0 ± 20.9% of the control by 1:1 and 1:3 without fibrin, respectively, and to 85.5 ± 34.3% by 3:1 with fibrin). As in the other cases, the effect was milder in the presence of fibrin (Figure 7C).

## 4. Discussion

Fibrin-based sealants commonly used in surgery are not only a biocompatible tissue adhesive but also a useful method of delivering biologically active compounds both at the site of use and at remote locations by releasing the drug into the blood or lymph. Even a small improvement in the quality of the obtained tissue joint may prove useful in reducing the production cost of the preparation by using lower concentrations of human or potentially immunogenic bovine thrombin or reducing the concentration of lysis inhibitors such as aprotinin [40]. Both the thromboelastometric analyses and the confocal microscopy showed a statistically significant influence of the S6 fraction on the physical parameters of the fibrin clot. The density of the fibrin clot as well as its cross-linking increased. Parameters related to the initiation of clot formation for concentrations from 30 µg/mL to 300 µg/mL were decreased in a dose-dependent manner, while parameters related to coagulation propagation were increased, compared with the control. Maximum clot firmness (MCF) and shear elastic modulus strength (G) increased in a dose-dependent manner. In the case of the S6 fraction in concentrations equal to and above 600 µg/mL, deterioration of the fibrin clot parameters responsible for clotting initiation, propagation, and stability was observed. This result may be explained by the fact that a decrease in thrombin activity was observed also at these concentrations. The decrease in proteolytic activity may be related to the presence of a non-competitive inhibitor of this serine protease in the tested fraction, manifesting itself when higher concentrations of S6 were used. This corresponds to the observation that the inhibitory effect of high S6 concentration (1000 µg/mL) may be abolished by increasing the amount of thrombin. Selective inhibitors of this enzyme are quite common among wood rot fungi [41]. Enhancement of thrombin activity by S6, which may have led to the above results, is rather unlikely since we did not observe elevated cleavage of the synthetic substrate for thrombin. A more probable mechanism of the observed augmentation of fibrin formation in the presence of S6 is the increase in polymerization and cross-linking of fibrin protofibrils.

Undoubtedly, this type of result will require more in-depth analyses of the interactions between molecules. Luo et al. analyzed the interaction of common phenolic acids (including p-hydroxycinnamic acid derivatives) with proteins of the blood coagulation cascade by determination of the binding energy of phenolics to coagulation factors and showed their pro- and anticoagulant properties [42]. The presence of phenolic compounds (including phenolic acids), which are common among the secondary metabolites of such wood rot fungi as *C. unicolor* [6,22,43,44], may be, although not exclusively, responsible for the procoagulant properties of the tested fraction.

Compared with the intense inhibition of cell proliferation by fraction S5 without the addition of fibrin, the activity of fractions S5 and S6 combined with the fibrin matrix was low, and a statistically significant result was only found at the concentration of 300 µg/mL for the mixture of fractions S5 and S6 in the ratio 3:1. A possible explanation for this is the proliferation-stimulating effect of thrombin, which can promote the proliferation of tumor cells such as HT-29 by acting on PAR-type receptors (protease-activated receptors) such as PAR-1 [45]. However, in the proposed test model, the control trial with the addition of pure thrombin (results not shown) did not indicate a statistically significant effect of thrombin. In addition, the bioavailability of the tested fractions may be limited by the slow release of the active substance from the protein matrix of the fibrin clot. Interestingly, Zhang et al. observed a positive effect of low-density salmon fibrin on the promotion of colorectal stem cell proliferation related to an increase in the Nanog self-renewal molecule. There was an inverse correlation between the proliferation and the density of the fibrin gel [46]. Similar correlation in the HT-29 line with the human fibrin matrix was also observed (Figure S2). Therefore, for in vitro analyses, the concentration of fibrinogen was selected to ensure the appropriate growth of the cell lines. This resulted in the promotion of cell proliferation even by preparations containing the S5 and S6 fractions. However, the combinations of the fractions, especially in the 1:1 and 1:3 proportions, exhibited distinct properties leading to a decrease in the proliferation of the HT-29 cells. This may indicate antagonistic action of the extracts or the presence of inhibitors. Noteworthy, samples administered without the fibrin matrix inhibited the proliferation of HT-29 to an extent comparable with what has been previously reported [22]. Fibrinogen and fibrinogen-like proteins have been demonstrated to induce cell proliferation through upregulation of a number of pathways and factors, including MAPK/ERK and Ki67 protein [47,48,49]. In the present study, the fractions at 100 µg/mL in combination with fibrinogen induced cell proliferation, while the lower and higher concentrations of the fractions abolished such activity. This may suggest high interaction of the compounds present in the fractions. This matter, however, needs further investigation.

The observations of morphological changes in the HT-29 cells treated with S5 and S6 without fibrinogen revealed that all variants were effective in apoptosis induction, compared with the results obtained from the normal CCD 841 CoTr cells, where no significant apoptotic nor necrotic effect was observed either with or without fibrin. The combinations of fractions were more efficient, and the 3:1 combination exhibited the strongest pro-apoptotic effect. We demonstrated that the fibrinogen addition seemed to exert a protective effect on cell cultures. This may be the effect of the in vitro cell culture limitations, which do not fully reflect the tumor microenvironment. Hu et al. revealed improvement in tumor apoptosis promotion, tumor angiogenesis inhibition, and a decrease in proliferation by an oxaliplatin and fibrin glue combination in vivo, compared with application of a single agent [26]. Additionally, Sanoj et al., showed that curcumin-loaded fibrinogen nanoparticles were comparatively nontoxic to a mouse fibroblast cell line (L929) but toxic to prostate (PC3) and breast (MCF7) cancer cells. Evaluation of apoptosis revealed increased apoptosis in MCF-7 compared with L929 cells in vitro [50].

Metalloproteinases of the extracellular matrix (MMPs) including MMP-9 (a gelatinase capable of digesting type IV collagen) play an important role in the process of colon carcinogenesis as well as in the wound healing process. The degradation of type IV collagen is found in both invasion and metastasis, and the expression of MMP-2 and MMP-9 is significantly higher in colon cancer cells [51,52,53]. In turn, excess as well as deficiency of MMP-9 and MMP-2 can have adverse effect on wound healing of normal tissues [54,55,56,57,58]. The estimation of the MMP-2 and MMP-9 levels in cell cultures with or without fibrin addition yielded interesting results. In both cases, <50 kDa isoforms of the MMP-9 protein were detected, and their expression level was decreased in almost every variant. This isoform was less prone to inhibition, which may lead to worse recovery prognosis of cancer patients [59]. At the same time, in the cell cultures with no fibrin application, both the single application and the combination of fractions decreased the MMP-2 and MMP-9 levels and activity. This may have a positive effect in the context of reducing metastasis and accelerating the healing of chronic wounds [52,56]. The pro-MMP-9 level was decreased as well. However, the level of pro-MMP-2 was increased in every variant, excluding S6. The interaction between MMP-2 and fibrinogen has been demonstrated before. Monaco et al. found that MMP-2 may catalyze fibrinogen cleavage [60]. In turn, Sarker et al. demonstrated that human fibrinogen may inhibit MMP-2 and pro-MMP-2 in a concentration-dependent manner [61]. The pro-MMP-2 level-increasing effect demonstrated in our study may be related to activation of the enzyme as a response to the presence of fibrinogen/fibrin in the environment. Nevertheless, the issue is to be addressed in the future.

The results of the use of fibrin supplemented with fungal bioactive fractions as a natural drug carrier are promising and further research, especially in vivo, is needed. There are several studies suggesting that fibrinogen glue may be used as a drug carrier [26,62,63]. In the present study, we demonstrated that natural fungal anticancer agents from *C. unicolor* not only may be released from the fibrinogen carrier but also may promote its polymerization.

In conclusion, enrichment of the fibrin sealant with the S6 and S5 fractions improves its structural and viscoelastic properties. Increasing the strength and cross-linking density of fibrin can not only reduce the cost of production and the potential immunogenicity associated with the use of bovine thrombin, or the potential complications associated with the use of lysis inhibitors, but can also improve the quality of the fibrin sealant. An increase in the cross-linking and thickness of fibrin fibers may also turn out to be beneficial when using fibrin glues/sealants as drug carriers, where the drug release time depends on the cross-linking nature of the carrier [64,65]. The effect of the S5 fraction on the stimulation of cell proliferation in the presence of fibrinogen is also important, as it points to its potential to promote wound healing. At the same time, the appropriate proportions of factors S5 and S6 can inhibit migration, moderating the activity of matrix metalloproteinases.

## 5. Conclusions

Summarizing, the presented results show the potential of the studied *Cerrena unicolor* low molecular weight fractions in combination with fibrinogen as a factor promoting wound healing and a natural biocompatible tissue binder. The analyzed factors can act both as compounds with antitumor and antimetastatic potential and as wound healing promoters after surgery. They can accelerate the formation of a fibrin clot and improve the physical properties of fibrin fibers of the surgical sealant [66]. There is no doubt that both studied fractions are an interesting research subject. The potential use in the process of postoperative wound healing and concomitant reduction of potential metastasis processes seems to be extremely valuable.

## Supplementary Materials

The following are available online at https://www.mdpi.com/article/10.3390/biom11091263/s1, Figure S1: The effect of S and S1–S6 subfractions on the clotting lag time in human plasma (A), clotting time (B), and clot formation rate (C) expressed as percentage of control was determined. Figure S2: Effect of the human fibrinogen on normal CCD 841 CoTr and cancer HT-29 cell (A) viability (NR method) and (B) proliferation (MTT method).
