# Supplementary material for: Thromboelastometric Analysis of Anticancer Cerrena unicolor Subfractions Reveal Their Potential as Fibrin Glue Drug Carrier Enhancers
Source: Biomolecules. 2021 Aug 24;11(9):1263. doi: 10.3390/biom11091263 (PMC8470457; doi:10.3390/biom11091263)
Supplement: Supplementary file 1 [file biomolecules-11-01263-s001.zip › biomolecules-1301993-supplementary.pdf]

Article

Thromboelastometric analysis of anticancer *Cerrena unicolor* subfractions reveal their potential as fibrin glue drug carrier enhancers

Supplementary:

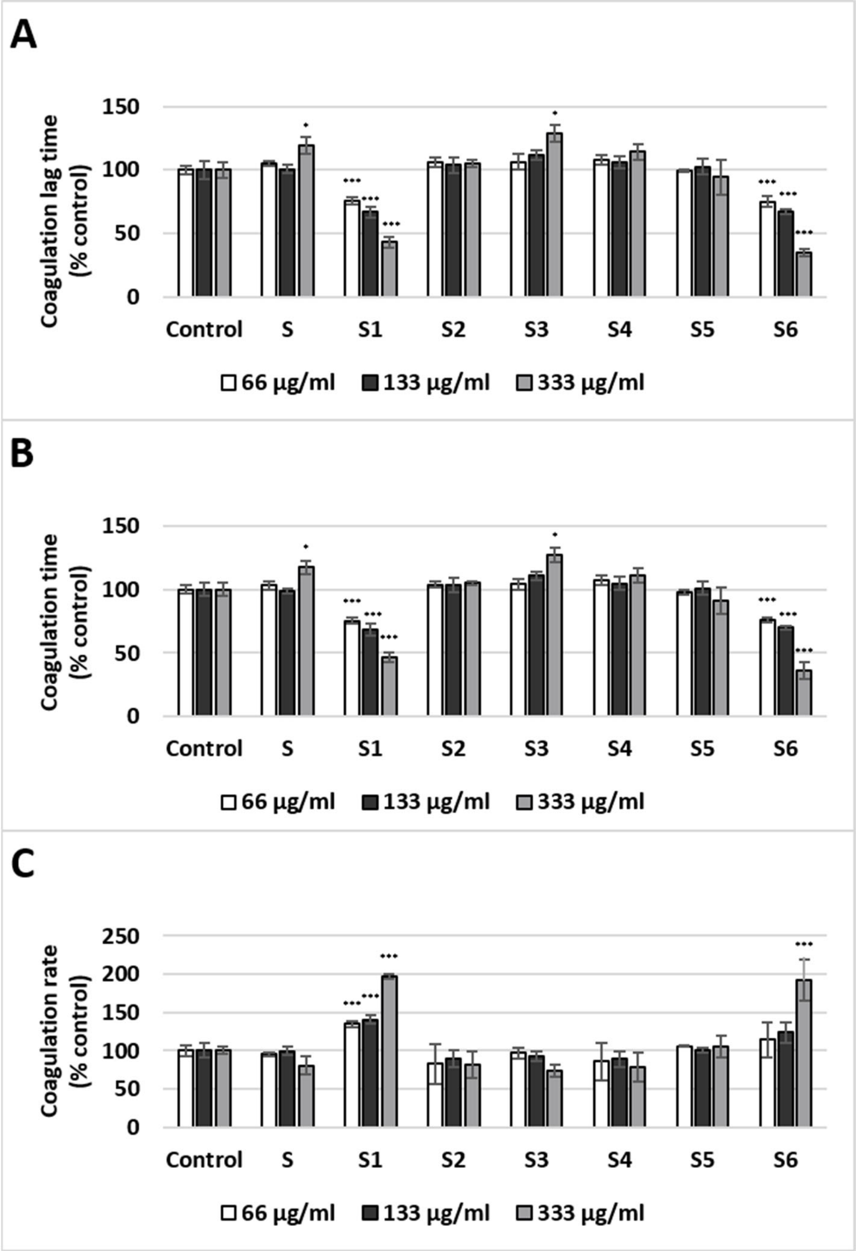

Figure S1. The effect of S and S1 - S6 subfractions on the clotting lag time in human plasma (A), clotting time (B), and clot formation rate (C) expressed as percentage of control was determined. The coagulation analysis was performed on human plasma, 25 µl of plasma per well was added to

microtiter plates. Then, a 50 µl Tris-HCl-NaCl pH 7.4 buffer (50 mM Tris-HCl, 100 mM NaCl) and 25 µl of test substances at final concentrations of 66, 133, and 333 µg/ml and saline as control were added. The final mixture was incubated at 37 °C for 10 minutes. Subsequently, the coagulation process was activated by adding 50 µl of 60 mM $CaCl_2$ in a Tris-HCl-NaCl buffer, taking into account the pipetting time in the final measurement [66]. The coagulation measurement was carried out for 30 minutes in 12-second cycles at 340 nm at 25 °C using a Tecan Sunrise plate reader (Tecan, Männedorf, Switzerland). The coagulation delay time was expressed as the number of seconds between the initiation of the process and the start of coagulation characterized by an increase in absorbance. The coagulation time was expressed as the absorbance increase from the start of coagulation until the maximum absorbance was reached. The coagulation rate was the absorbance increase per second (AU/s) between the minimum and maximum absorbance. Values are presented as the mean of triplicates with standard deviation and test probability (*** p> 0.005; ** p> 0.01; * p> 0.05).

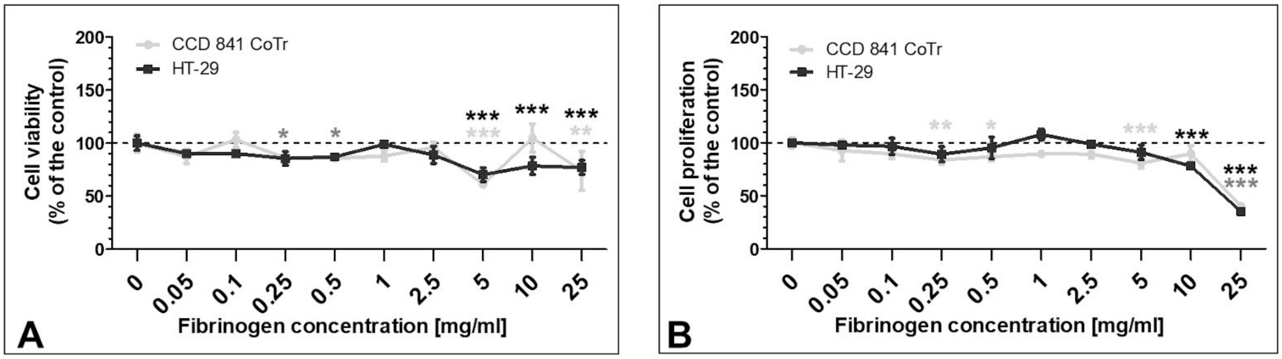

Figure S2. Effect of the human fibrinogen on normal CCD 841 CoTr and cancer HT-29 cell (A) viability (NR method) and (B) proliferation (MTT method). * - p < 0.05; * - p < 0.01; *** - p < 0.005; one-way ANOVA, post-hoc: Dunnett's test.
